# Editorial: Managing Parkinson's Disease With a Multidisciplinary Perspective

**DOI:** 10.3389/fneur.2021.799017

**Published:** 2021-11-26

**Authors:** Seyed-Mohammad Fereshtehnejad, Mayela Rodríguez-Violante, Daniel Martinez-Ramirez, Adolfo Ramirez-Zamora

**Affiliations:** ^1^Division of Neurology, Department of Medicine, University of Ottawa, Ottawa, ON, Canada; ^2^Division of Clinical Geriatrics, Department of Neurobiology, Care Sciences and Society (NVS), Karolinska Institutet, Stockholm, Sweden; ^3^Movement Disorders Clinic, Clinical Neurodegenerative Research Unit, Mexico City, Mexico; ^4^Tecnologico de Monterrey, Escuela de Medicina y Ciencias de la Salud, Monterrey, Mexico; ^5^Department of Neurology, Fixel Institute for Neurological Diseases, University of Florida, Gainesville, FL, United States

**Keywords:** multidisciplinary approach, treatment, interdisciplinary teams, patient-centered care, management, Parkinson's Disease (PD)

Parkinson's Disease (PD) is a complex and heterogeneous disorder from prodromal to advanced stages, with a wide range of motor and non-motor symptoms from bradykinesia and rigidity to dysautonomia, sleep disturbances, and cognitive impairment (1). Such a clinical diversity necessitates the involvement of healthcare professionals from different disciplines for an optimal management plan. Clinical guidelines recommend that all persons with PD should have access to a broad range of medical and allied health professionals for personalized, integrative, and multidisciplinary care (2, 3). This is supported by growing evidence showing the effects of integrated multidisciplinary interventions on improving quality of life and disease progression in people with PD (4–6).

Apart from symptomatic management and clinical care, other aspects of PD also warrant a multidisciplinary approach with a wide variety of expertise being involved from discovery of its pathophysiology and early prodromal stage to progression of parkinsonian features as medications' side effects. Roiter et al. analyzed the prevalence of parkinsonian symptoms in patients with severe mental illnesses admitted to a monocentric inpatient psychiatry ward. Parkinsonian (previously referred to as extrapyramidal) symptoms were quite common in this population (50.5%), with tremor (33%) being the most common manifestation. Only a minority of patients met the criteria for possible PD, which were significantly older, had more medical comorbidities with a larger number of medications including antipsychotics, and slightly more than half of them had reduced striatal dopamine transporter binding in ^123^I-FP-CIT SPECT. Whether antipsychotic medications unmask underlying neurodegenerative pathology by quickening the progression of the prodromal stage is not yet clear (7).

Gastrointestinal problems such as constipation are increasingly appreciated as another non-motor prodromal feature (1) and/or risk factor for developing PD (8), integrating another discipline into the diagnosis, and care of, people with PD. In their longitudinal study on *de novo* PD patients, Palacios et al. showed that Levodopa, as the mainstay of PD symptomatic therapy, did not change the gut microbiota composition after 3 months of initiation. Nevertheless, they observed a marginally lower abundance of *Clostridium* group IV in PD patients with a good response to Levodopa, which needs further investigation in the future.

Multidisciplinary diagnostic approaches such as neuroimaging techniques and other biomarkers are helpful to analyze overlapping movement disorders syndromes. There is evidence suggesting that patients diagnosed with essential tremor (ET) are at increased risk of developing PD (9). This relationship has been controversial as both disorders are common with significant clinical overlap between them. In this special article, Wang et al. reviewed the evidence on the application of neuroimaging [i.e., transcranial sonography (TCS), voxel-based morphometry, diffusion tensor imaging, proton MR spectroscopy], and potential multidisciplinary clinical biomarkers (i.e., non-motor symptoms and heart rate variability) suggestive of prodromal PD in ET. Current evidence suggests that patients with ET have an increased risk of developing PD, especially those with substantia nigra hyperechogenicity in TCS and in ET patients with specific tremor features, non-motor, genetic, and autonomic characteristics.

One very well-appreciated multidisciplinary aspect of PD is the caring and management practices beyond the routine protocols. There is an increasing interest in assessing the benefits of different physiotherapy and exercise interventions in patients with PD. Multiple mobility and rehabilitative strategies have been recognized as part of a multidisciplinary approach to PD management. As such the roles of allied health care members, namely physiotherapists and occupational therapists, in the multidisciplinary care of PD have been well-recognized (10). In a small, open-label, prospective study (Kotani et al.) aimed to investigate whether core exercise complemented with a hybrid assistive limb for lumbar support may improve the motor function in PD patients. The authors reported significant improvement in standard, validated motor measures at the end of the trial (after 3 months) with adequate safety. In a randomized controlled clinical trial, Segura et al. assessed the impact of a high-intensity tandem bicycle program on symptoms severity. They noted significant improvement in motor function clinically and in biochemical and functional neuroimaging variables. Giménez-Llort et al. proposed the “PasoDoble,” as a novel dance/music patient-caregiver intervention in PD patients. In this methods article, they reviewed the rationale on evidence-based therapeutic benefits of dance/music therapy and highlighted the potential benefit of this specific multidisciplinary technique.

Complementary medicine involves health care practices not considered as part of standard care that are used along with conventional medicine; when these practices are combined in a coordinated and standardized manner the result is integrative medicine (11). Huang et al. performed a systematic review on the effectiveness of acupuncture using the AMSTAR-2 and GRADE criteria. Overall, acupuncture was safe and improved some axial parkinsonian symptoms. However, the more striking finding was that most studies were rated as very low to low quality thus highlighting the need for better-designed studies in the future. Sharpe et al. reviewed non-pharmacological interventions in the context of axial and cognitive symptoms in early PD. The effect of balance, posture, speech, swallowing, and cognitive interventions proved to be of value highlighting the role of a unified multidisciplinary plan with an individualized approach.

Advanced management in PD, as another aspect of multidisciplinary care, is usually needed when oral or transdermal medications fall short in controlling the motor and non-motor fluctuations and accompanying complications such as dyskinesia with a large impact on quality of life and daily life activities (12). These advanced therapies include deep brain stimulation (DBS), continuous levodopa-carbidopa intestinal gel, and continuous subcutaneous apomorphine infusion. Qi et al. reported motor outcomes at 1 year in 40 individuals with PD who underwent subthalamic nucleus DBS. DBS improved motor function in all patients with various indexes of progression, yet the fast-progression group had less improvement. The slightly different outcome depending on the preoperative progression index might be a useful indicator for the multidisciplinary team involved in this type of management.

Aye et al. conducted a review with the objective of presenting current evidence and gaps in knowledge of multidisciplinary management and provision of community care of people with PD. The authors described their 14-year experience based on the Partners Community Care Program in Singapore that provides care for people with PD in the community and at their homes. The authors argued that although there is scarce experimental evidence supporting the use of multidisciplinary management, probably due to the great heterogeneity of the interventions and the need to individualize treatment, multidisciplinary management in PD should be recommended. Interdisciplinary or transdisciplinary support, as well as remote support with tools such as telemedicine, are feasible methods to provide and achieve the ideal patient-centric management. In their perspective article, Göttgens et al. discussed the impact of “sex” and “gender” in the multidisciplinary management of patients with PD. For motor symptoms such as imbalance and dyskinesias, authors propose possible sex- and gender-sensitive interventions such as balance training and DBS. Regarding non-motor symptoms, a reduction or discontinuation of dopaminergic therapies, cognitive behavioral therapy, referrals for training coping skills, and social support interventions were suggested. With respect to lifestyle, authors recommend self-monitoring of weight and dietetic plan for men and physical exercise and motivational interventions in women. Finally, in terms of social support, they suggest proactively identifying patients' social networks and referral to social support groups as well as regular evaluations to identify caregiver strain and improve their education about PD. It is crucial to identify these sex- and gender-related differences in knowledge and care provision to optimize the multidisciplinary management plan for people with PD.

Capitalizing on the importance of managing PD with a multidisciplinary approach, one must consider its potentially prohibitive cost by involving a broad range of medical and allied health professionals. One way to overcome its financial burden is innovative virtual or in-person platforms such as online health communities or patients' networks where professionals and patients can meet online in a secured environment to share their experiences (10).

## Author Contributions

This editorial was written by S-MF, MR-V, DM-R, and AR-Z and edited by S-MF. All authors contributed to the article and approved the submitted version.

## Conflict of Interest

The authors declare that the research was conducted in the absence of any commercial or financial relationships that could be construed as a potential conflict of interest.

## Publisher's Note

All claims expressed in this article are solely those of the authors and do not necessarily represent those of their affiliated organizations, or those of the publisher, the editors and the reviewers. Any product that may be evaluated in this article, or claim that may be made by its manufacturer, is not guaranteed or endorsed by the publisher.
